# Biocontrol Potential of Native Entomopathogenic Bacteria Against *Palpita persimilis* in Peruvian Olive Agroecosystems

**DOI:** 10.3390/plants15121786

**Published:** 2026-06-10

**Authors:** Angela Verónica Choque Miranda, César Julio Cáceda Quiroz, Milena Carpio Mamani, Gisela July Maraza Choque, Niccol Milagros Paredes Jahuira, Jorge González Aguilera, Hebert Hernán Soto Gonzales

**Affiliations:** 1 Bioremediation Laboratory, Science Faculty, Universidad Nacional Jorge Basadre Grohmann, Av. Miraflores S/N, University City, Tacna 23003, Perumcarpiom@unjbg.edu.pe (M.C.M.);; 2 Department of Agronomy, Cassilândia Campus, State University of Mato Grosso do Sul (UEMS), Cassilândia 79543-113, Brazil; 3 Laboratorio de Biología Molecular y Biotecnología, Escuela Profesional de Ingeniería Ambiental, Universidad Nacional de Moquegua, Ilo 18601, Peru

**Keywords:** biological control, *Olea europaea* L., olive pests, rhizospheric bacteria

## Abstract

Olive, *Olea europaea* L. (Oleaceae), cultivation is affected by significant yield losses caused by *Palpita persimilis* Munroe (Lepidoptera: Crambidae), a defoliating pest in South America. Its control currently relies on synthetic pesticides, which have adverse environmental effects. This study investigated native entomopathogenic bacteria isolated from the rhizosphere of olive trees in Tacna, Peru. A total of 36 bacterial isolates were obtained, of which six strains showing more than 70% larval mortality were selected for further evaluation. Morphological and molecular analyses suggested a tentative affiliation of the isolates with bacterial groups related to the genera *Lysinibacillus*, *Paenibacillus*, *Priestia*, and *Bacillus*. Bioassays demonstrated that larval mortality depended on the bacterial concentration and exposure time. Strains such as *Peribacillus* sp. UNM achieved 100% larval mortality after 96 h at a concentration of 1 × 10^9^ CFU mL^−1^. Analysis using a generalized linear model (GLM) with a binomial distribution confirmed that bacterial strain, concentration, and exposure time significantly influenced larval mortality, indicating that mortality responses varied according to bacterial concentration and exposure time. These findings provide preliminary laboratory evidence of entomopathogenic activity associated with native bacterial isolates against *P. persimilis* and support future investigations aimed at evaluating these isolates under field conditions in olive agroecosystems.

## 1. Introduction

Olive, *Olea europaea* L. (Oleaceae), cultivation is an agricultural activity of major economic importance and is primarily concentrated in the Mediterranean region [1]. Currently, more than 11.1 million hectares are cultivated worldwide, with the largest proportion in Europe (64.4%), followed by Africa (16.9%), Asia (15.7%), and the Americas (2.7%) [2]. Although Spain and Italy have led global production, the expansion of olive cultivation into non-traditional regions has been driven by the sustained increase in global demand for olive oil and table olives over the past two decades, promoting its establishment in Southern Hemisphere countries such as Argentina, Chile, and Peru [3]. In South America, the harvested area reaches 182,818 ha, with a total production of 678,606.7 t reported in 2024 [2]. In Peru, approximately 28,300 ha is cultivated, with a production of 229,698.3 t, representing approximately 15% of total South American production [4]. The Tacna region is the main olive-producing area in the country, with a production of 176,423 tons across 19,695 ha in 2022 [5].

Olive cultivation faces multiple phytosanitary challenges, which are being exacerbated by climate change, increasing the incidence, severity, and geographic distribution of agricultural pests [6,7]. In the Mediterranean region, *Bactrocera oleae* and *Prays oleae* are the predominant pests. In contrast, in South America, the main pests include olive scale (*Saissetia oleae*), moths of the genus *Palpita* spp., and the mite (*Oxycenus maxwelli*) [6,7]. In the Tacna region, olive groves are particularly affected by the olive shoot worm *Palpita persimilis* Munroe (Lepidoptera: Crambidae), the flower looper (*Cyclophora serrulata*), the bark beetle (*Phloeotribus scarabaeoides*), and the mobile scale (*Orthezia olivicola)* [8].

The genus *Palpita* Hübner comprises moth species with tropical and temperate distributions, many of which are associated with plants of the family Oleaceae [9]. In particular, species within this genus can cause severe defoliation, affecting up to 90% of the foliar area and reducing crop yield by approximately 30%, especially during critical stages of fruit development [10]. These species are considered major pests of olive and jasmine crops in the Mediterranean Basin and Asia [11,12,13,14]. Other economically relevant species include *Palpita nigropunctalis*, as well as *Palpita forficifera* and *P. persimilis*, which cause fruit damage in mature trees and floral and foliar deformation in young trees [6,15,16]. Their control is particularly challenging due to the cryptic feeding behavior of larvae, which feed within silk shelters, leading to severe defoliation, reduced photosynthetic area, loss of vegetative vigor, shoot deformation, and direct yield losses [14,17].

The intensive use of synthetic pesticides has generated significant ecological and economic impacts [18,19], highlighting the urgent need to implement agroecological strategies and integrated pest management (IPM) approaches to mitigate these effects [20,21]. In this context, microbial biocontrol has emerged as a sustainable alternative with increasing scientific and commercial interest because of its ability to reduce reliance on chemical inputs [22,23,24], while providing an effective approach to suppress pest populations and limit crop damage [25,26].

Entomopathogenic bacteria (EPB) and their toxins are among the most successful microbial insecticides commercially available [27]. These organisms exert their effects through the production of insecticidal secondary metabolites or enzymes that disrupt essential physiological processes [28,29]. They also damage the midgut epithelium of insects, facilitating bacterial invasion into the hemocoel, followed by systemic infection and septicemia, ultimately leading to host death [30,31]. Among EPB, *Bacillus thuringiensis* (Bt) is the most widely used and effective species against pests of the orders Lepidoptera, Coleoptera, and Diptera [32]. Bt has demonstrated efficacy against *Spodoptera frugiperda*, *Musca domestica*, *Helicoverpa armigera*, *Spodoptera cosmioides*, and *Spodoptera eridania* [33,34,35,36]. However, its effectiveness against species of the genus *Palpita*, including *P. persimilis*, has rarely been documented and has shown variable outcomes depending on the species and experimental conditions [12]. Additionally, the emergence of resistance to Bt in lepidopteran populations underscores the need to identify new sources of EPB with insecticidal properties [37,38,39].

Other EPB, such as *Paenibacillus popilliae*, *Brevibacillus laterosporus*, and *Serratia entomophila*, are commercially available for the control of coleopteran and lepidopteran pests, although they represent only a small fraction of the global EPB market [40]. Overall, bacterial biopesticides dominate the biopesticide sector, accounting for approximately 74% of the global market, followed by fungal (10%), viral (5%), and nonmicrobial alternatives (11%) [29,41].

Nevertheless, despite their relevance within the biopesticide sector, entomopathogenic bacteria represent only 1.9% of the global pesticide market, although they account for approximately 66% of registered microbial products, highlighting a discrepancy between scientific development and large-scale implementation [40,42]. While native entomopathogenic strains have been reported from olive leaves in northwestern Egypt [12], the rhizosphere remains an underexplored reservoir of bacteria with entomopathogenic activity, particularly in olive agroecosystems in South America and Peru. This lack of experimental evidence represents a critical knowledge gap that limits the development of targeted and locally adapted biocontrol strategies.

Furthermore, there is limited understanding of the specific susceptibility of *P. persimilis* to different genera of EPB, as well as the variability in larval responses under controlled conditions. Although previous studies have evaluated EPB in olive systems in Mediterranean regions, extrapolation of these findings to South American agroecosystems is constrained by climatic, edaphic, and ecological differences. The objective of this study was to evaluate the entomopathogenic activity of native bacteria isolated from the olive rhizosphere in southern Peru against larvae of *P. persimilis* through bioassays conducted under controlled conditions.

## 2. Results

### 2.1. Isolation of Entomopathogenic Bacteria from Native Soils

A total of 36 bacterial strains were isolated from rhizospheric soil samples collected from olive cultivation systems (Table 1). Approximately 80% of the isolates corresponded to Gram-positive bacilli, whereas the remaining 20% included Gram-negative bacilli and Gram-positive cocci.

From the preliminary screening, 15 isolates exhibiting more than 50% larval mortality were selected, of which six were further evaluated at three concentrations. These six isolates (2M-5, 3M-19, 4M-1, 4M-14, 4M-26, and 6M-18) were subjected to subsequent bioassays, achieving larval mortality rates exceeding 70%.

**Figure 1:**
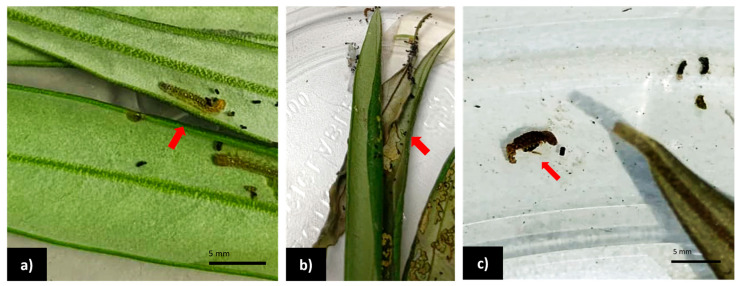
Effects associated with exposure to *Lysinibacillus* sp. ACM on *P. persimilis* under laboratory conditions. (**a**) Healthy third-instar larva (L2), (**b**) visible leaf tissue damage with the presence of frass and cuticular remnants, and (**c**) dead larva after exposure to the experimental bacterial treatment via spray application.

These results indicate considerable variability in entomopathogenic efficacy among the isolates. Pathogenicity assays demonstrated larvicidal activity of *Lysinibacillus* sp. ACM against larvae of *P. persimilis* (Figure 1). Healthy larvae (Figure 1a) exhibited uniform coloration and active feeding behavior on untreated olive leaves. In contrast, larvae exposed to bacterial isolates (Figure 1a) showed damage to leaf tissue and altered feeding patterns. High mortality was subsequently observed (Figure 1c), characterized by immobility and morphological changes such as body curvature and tissue darkening. These observations support the entomopathogenic activity of the evaluated isolates.

### 2.2. Morphological and Microscopic Characterization of Bacteria Isolated from Native Soils

The six bacterial isolates were selected based on their entomopathogenic activity. Macroscopic and microscopic observations revealed notable morphological variation among the isolates (Table 2). All strains formed large colonies (2–4 mm) with cream to beige pigmentation, dry texture, irregular margins, and an opaque appearance. Endospore formation was observed in all isolates, a characteristic commonly reported in bacterial groups related to *Priestia* and *Lysinibacillus*.

### 2.3. Molecular Identification

The tentative taxonomic assignment of native bacterial strains with entomopathogenic activity was based on BLAST (https://blast.ncbi.nlm.nih.gov/Blast.cgi, accessed on 1 February 2026) analysis of partial 16S rRNA gene sequences (~600 bp) obtained from strains 2M-5, 3M-19, 4M-1, 4M-14, 4M-26, and 6M-18. Although BLAST results showed relatively low sequence identity and query coverage (<95%) compared with reference sequences available in GenBank, the closest matches suggested affinity with bacterial groups related to *Priestia*, *Lysinibacillus*, *Paenibacillus*, and *Peribacillus*. Phylogenetic analyses revealed broad clustering patterns, suggesting tentative placement of strains 2M-5 and 4M-26 within *Priestia*, strain 3M-19 within *Paenibacillus*, strains 4M-1 and 4M-14 within *Lysinibacillus*, and strain 6M-18 within *Peribacillus* (Table 3).

However, the low sequence identity and query coverage values observed for several isolates, particularly strains 4M-1 and 2M-5, substantially limit the robustness of taxonomic inference. Therefore, the phylogenetic relationships observed in this study should be interpreted cautiously and considered exploratory rather than definitive evidence of taxonomic placement.

Owing to the limited resolution provided by partial 16S rRNA gene sequences, the taxonomic identity of these strains is considered putative. Therefore, complementary approaches, including whole-genome sequencing, are currently being undertaken to achieve more robust and definitive classification.

The phylogenetic analysis was performed using the Maximum Likelihood (ML) method under the Tamura–Nei substitution model, implemented in MEGA version 12.0.15. Two phylogenetic trees were constructed based on 16S rRNA gene sequences: a general tree including 56 sequences comprising native strains and type strains retrieved from GenBank, and a second tree specific to strain 4M-1, which included 102 representative sequences to further explore its phylogenetic relationships.

In both analyses, *Escherichia fergusonii* was used as an outgroup to root the trees. The robustness of the inferred groupings was evaluated using bootstrap analysis with 1000 replicates, and the corresponding values are shown at each node.

**Figure 2:**
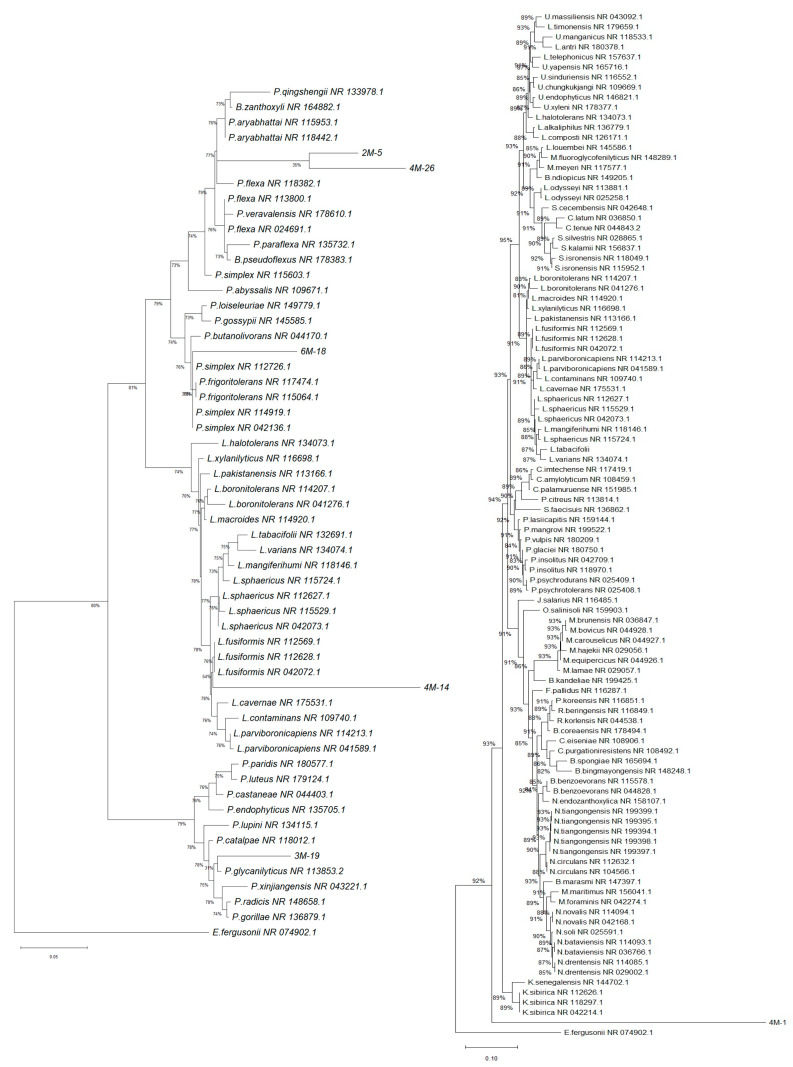
Phylogenetic tree based on partial 16S rRNA gene sequences of the 6 bacterial strains selected for the entomopathogenic bioassays (MEGA v. 12.0.15).

The phylogenetic analyses revealed broad clustering patterns of native strains 2M-5, 3M-19, 4M-1, 4M-14, 4M-26, and 6M-18 within the *Bacillus sensu lato* clade, showing affiliations with phylogenetic groups related to the genera *Priestia*, *Paenibacillus*, *Peribacillus*, *Bacillus* and *Lysinibacillus* (Figure 2). However, owing to the limited resolution of partial 16S rRNA sequences, whole-genome sequencing would be necessary to further explore the phylogenetic relationships of these isolates.

### 2.4. Laboratory-Scale Entomopathogenic Activity Assay

During the pathogenicity bioassays, no mortality was observed in the negative control throughout the experimental period (24, 48, 72, and 96 h), indicating that the experimental conditions did not induce significant larval mortality in the absence of bacterial exposure.

**Figure 3:**
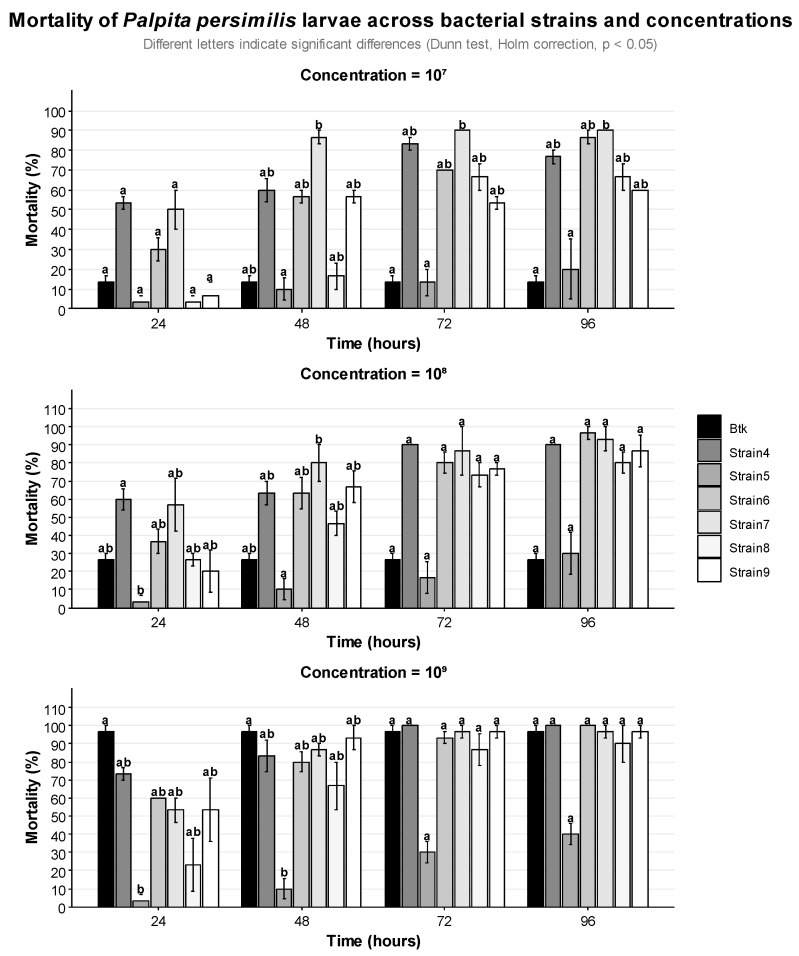
Larval mortality of *P. persimilis* exposed to different bacterial strains at varying concentrations. Each treatment (strain × concentration) consisted of three independent replicates, with 10 larvae per replicate (*n* = 30 larvae per treatment). Mortality was assessed at 24, 48, 72, and 96 h post-exposure. Bars represent the mean ± standard error of three replicates. Different letters above the bars indicate significant differences among strains at each exposure time, according to Dunn’s post hoc test with Holm correction (*p* < 0.05).

Mortality values (Table 4), together with the responses observed in Figure 3, indicated a progressive increase with inoculum concentration and exposure time, revealing concentration-dependent mortality responses. Entomopathogenic responses varied among strains in both magnitude and temporal progression. At the highest concentration (1 × 10^9^ CFU mL^−1^), *Peribacillus* sp. UNM and *Paenibacillus* sp. GCM reached 100 ± 0% mortality at 96 h, followed by *Lysinibacillus* sp. YLP and *Priestia* sp. JBG, which achieved 96.67 ± 3.33% mortality at the same time point.

At lower concentrations (1 × 10^7^ CFU mL^−1^) and during the initial 24 h of exposure, *Peribacillus* sp. UNM (60 ± 5.77%) and *Lysinibacillus* sp. YLP (50 ± 10%) exhibited the highest mortality rates. From 48 h onward, most strains exceeded 50% mortality as exposure time and inoculum concentration increased. These findings highlight substantial inter-strain variability and suggest that entomopathogenic efficacy was influenced by both concentration and exposure time.

The data in Figure 3 reveal significant differences among bacterial strains at exposure times earlier than 96 h and at concentrations below 1 × 10^9^ CFU mL^−1^ (*p* < 0.05), indicating variability in larval mortality among treatments under specific experimental conditions. In contrast, no significant differences among strains were detected at 96 h under the highest bacterial concentration (Kruskal–Wallis: χ^2^ = 11.859, df = 6, *p* = 0.065). In general, greater variability among strains was observed during the initial stages of exposure, whereas mortality responses became more homogeneous at later exposure times and higher bacterial concentrations. Several strains reached mortality values above 80% after 72–96 h of exposure, particularly at intermediate and high bacterial concentrations.

The generalized linear model (GLM) with a binomial distribution and logit link function showed a substantial improvement in model fit, reducing deviance from 1098.64 (null deviance) to 254.26 (residual deviance; df = 207; AIC = 684.50), enabling simultaneous evaluation of the effects of bacterial concentration (log_10_), exposure time, and strain on larval mortality of *P. persimilis*.

Likelihood ratio tests (Type II) indicated that bacterial concentration (χ^2^ = 92.85, df = 1, *p* < 0.001), exposure time (χ^2^ = 325.55, df = 1, *p* < 0.001), and strain identity (χ^2^ = 527.53, df = 5, *p* < 0.001) significantly affected larval mortality. Additionally, the interaction effect between concentration and time was significant (χ^2^ = 3.85, df = 1, *p* = 0.049), indicating that the effect of concentration varied according to exposure time (Table 5).

The logarithmic increase in inoculum concentration was associated with a significant increase in larval mortality (β = 0.36 ± 0.16, OR = 1.44, *p* = 0.021). In contrast, exposure time did not have a significant independent effect (*p* = 0.854). However, the interaction between concentration and time was marginally significant (β = 0.005, *p* = 0.051), indicating that mortality responses varied according to exposure duration under different bacterial concentrations.

Regarding bacterial strains (taking Strain4 as the reference), significant differences were observed among treatments. In particular, Strain5 showed a marked reduction in efficacy (OR = 0.03, *p* < 0.001), followed by Strain8 (OR = 0.29, *p* < 0.001) and Strain9 (OR = 0.49, *p* < 0.001), indicating lower larvicidal capacity compared to the reference strain. In contrast, Strain6 (*p* = 0.114) and Strain7 (*p* = 0.096) did not show statistically significant differences (Table 6).

Larval mortality was influenced by the bacterial strain, inoculum concentration, and exposure time, with higher mortality responses observed at intermediate and high concentrations after prolonged exposure periods. Collectively, the generalized linear model (GLM) results demonstrated that the entomopathogenic response was determined by the combined effects of these experimental factors.

**Figure 4:**
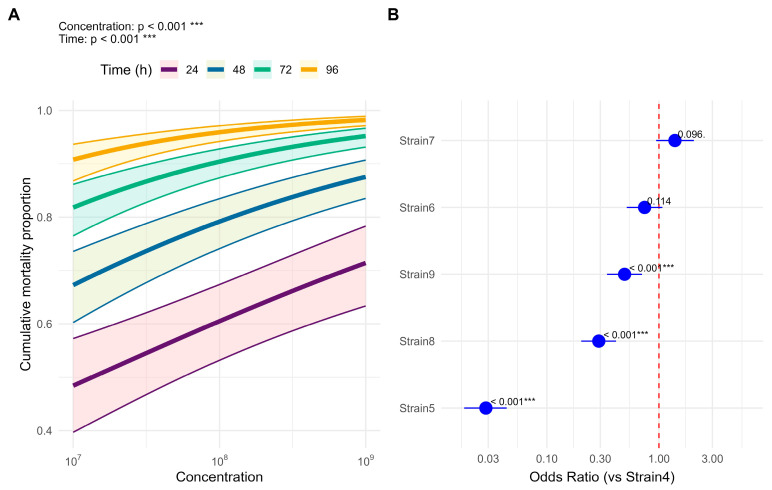
Predicted larval mortality of *P. persimilis* using a binomial generalized linear model (GLM) as a function of concentration, exposure time, and bacterial strain. In (**A**), lines represent the predicted probabilities of mortality. In (**B**), points denote odds ratios (ORs) and horizontal bars indicate 95% confidence intervals. An OR = 1 indicates no relative effect. Asterisks indicate levels of statistical significance: *** *p* < 0.001.

The predictive model incorporated the effects of concentration (logConc), time (Time), strain (Strain), and the logConc × Time interaction. The variation in larval mortality of *P. persimilis* as a function of bacterial strain, inoculum concentration, and exposure time is shown in Figure 4. Overall, a progressive increase in mortality was observed with increasing time and bacterial concentration.

In Figure 4A, larval mortality curves predicted by the binomial GLM revealed a consistent pattern of increasing mortality as a function of both inoculum concentration and exposure time. The probability of mortality increased progressively with increasing bacterial concentration, which is consistent with the positive and statistically significant effect of concentration identified in the model (*p* = 0.021). Although exposure time did not have a significant independent effect, the temporal trajectories were consistent with the marginally significant interaction between concentration and time (*p* = 0.051).

In Figure 4B, the adjusted odds ratios estimated by the GLM are presented for each bacterial strain relative to Strain4. Consistent with the model estimates, Strain5, Strain8, and Strain9 exhibited significantly lower larvicidal efficacy, whereas Strain6 and Strain7 did not differ significantly from the reference strain. These findings indicate that differences among bacterial strains contributed substantially to the variability observed in entomopathogenic efficacy among treatments.

Collectively, the GLM predictions (Figure 4A) and adjusted effect estimates (Figure 4B) indicate that larval mortality was primarily associated with bacterial concentration, while differences among bacterial strains and exposure time also influenced the entomopathogenic response.

## 3. Discussion

The evidence presented here indicates the presence of entomopathogenic bacteria (EPB) in the rhizospheric soils of *O. europaea* in southern Peru, putatively affiliated with bacterial clades belonging to the families Bacillaceae, Planococcaceae, and Paenibacillaceae. The predominance of Gram-positive bacilli may be attributed to their ability to form endospores, which confer enhanced resistance to adverse environmental conditions, as well as to their widespread distribution in soil and rhizospheric environments. Within this group, members of the family Bacillaceae were particularly prominent, representing dominant and naturally occurring components of the soil microbiome.

The exploration of native EPB in agroecosystems is relevant because it enables the identification of microorganisms adapted to local environmental conditions that could be evaluated in future biological control studies [43,44,45]. In addition, these soil-associated bacteria exhibit biocidal activity through multiple mechanisms, including the production of toxins, hydrolytic enzymes, bacteriocins, siderophores, antibiotics, extracellular enzymes, and endospore formation [30,46,47,48,49]. These mechanisms can lead to insect death either through septicemia [50] or via bacterial proliferation in the insect midgut following ingestion [51].

The strains evaluated in this study originated from a poorly explored arid agroecosystem. Most previous studies involving these bacterial genera (Table 7) have focused on other lepidopteran species, such as *Spodoptera*, *Plutella*, and *Helicoverpa*, whereas experimental evidence involving *P. persimilis* remains limited and has primarily been generated in Mediterranean regions and North Africa, including Egypt. In South America, available information is largely restricted to occurrence reports and observational records rather than experimental studies evaluating larval mortality associated with entomopathogenic bacteria [52,53]. Therefore, the present findings may be particularly relevant given the limited global information currently available on entomopathogenic mortality assays against *P. persimilis*.

The GLM analyses indicated that larval mortality was influenced by the combined effects of bacterial concentration, exposure time, and differences among bacterial strains under the evaluated laboratory conditions. In particular, the interaction between concentration and exposure time suggests that mortality responses vary according to both bacterial concentration and exposure duration. The higher mortality responses observed at intermediate and high bacterial concentrations after prolonged exposure periods indicate that the entomopathogenic response was progressive rather than immediate, with mortality increasing over longer exposure periods and at higher bacterial concentrations. Similar concentration- and exposure-dependent response patterns have previously been reported for entomopathogenic bacteria associated with the genera *Bacillus*, *Lysinibacillus*, and *Paenibacillus* in other lepidopteran species [69].

Differences detected among bacterial strains suggest variability in entomopathogenic responses among native isolates under similar experimental conditions. Such variability may reflect physiological differences among bacterial strains, although the mechanisms potentially associated with larval mortality were not evaluated in the present study. In agreement with the multivariable trends identified by the GLM, the exploratory analyses based on the Kruskal–Wallis test also revealed variability in larval mortality responses among bacterial strains under specific concentrations and exposure conditions. Collectively, these findings provide preliminary laboratory evidence for future investigations involving native bacterial isolates in the management of *P. persimilis*.

Among the evaluated isolates, *Peribacillus* sp. UNM (Strain4) demonstrated rapid activity, achieving 60% mortality at the lowest concentration (1 × 10^7^ CFU mL^−1^) within 24 h and 100% mortality at 96 h at the highest concentration (1 × 10^9^ CFU mL^−1^) against *P. persimilis*. These values are equal to or exceed those in previous reports for species of the genus *Peribacillus*, such as *P. simplex*, which achieved only 33% mortality in *G. mellonella* [55], and *P. frigoritolerans*, which showed variable mortality (74–89%) in larvae of *H. longipennis* and *A. dimidiata* [70]. Additionally, 45% mortality has been reported in *Agriotes lineatus* larvae after 10 days of exposure [71]. Although studies on lepidopterans are limited, this genus is widely recognized for its role in pesticide degradation and phytopathogen biocontrol [72,73].

Similarly, *Priestia* sp. JBG (Strain9) achieved high mortality (93.33%) at a concentration of 1 × 10^9^ CFU mL^−1^ within 48 h. These results are consistent with those reported for *Priestia megaterium* (formerly *B. megaterium*), whose mortality has reached 100% in *P. citrella* larvae after 144 h of exposure [58]. This genus has been associated not only with entomopathogenic activity but also with important agrobiotechnological functions, including polymer degradation [74], pesticide detoxification [64], industrial enzyme production [75], and plant growth promotion [76]. Furthermore, recent studies have demonstrated the environmental stability of insecticidal proteins, such as Vip3Ag4 expressed in *P. megaterium*, through microencapsulation strategies, highlighting their potential for use in biocontrol applications [59].

In the case of *Lysinibacillus* sp., Abreo et al. [67] reported that *L. xylanilyticus* induced 66% mortality in first-instar larvae of *A. sphaleropa* fed on diets treated with spore suspensions after 168 h of exposure. In contrast, in the present study, *Lysinibacillus* sp. YLP (Strain7) achieved 86.67% mortality against *P. persimilis* at a concentration of 1 × 10^7^ CFU mL^−1^ after 48 h of treatment, indicating that the activity was relatively high under the evaluated laboratory conditions. The entomopathogenic activity previously reported for bacteria associated with this genus has been related to the production of bioactive compounds and other virulence-associated factors capable of affecting physiological processes in insects [60,77,78,79]. However, these mechanisms were not evaluated in the present study and therefore should be interpreted cautiously within the context of the existing literature.

Although *Lysinibacillus sphaericus* has long been associated primarily with mosquito control [80], reports of its activity against lepidopteran species remain limited.

*Paenibacillus* sp. GCM (Strain6) also exhibited strong performance, achieving mortality rates above 90% after 72 h at a concentration of 1 × 10^9^ CFU mL^−1^, exceeding previously reported values for *Paenibacillus elgii* HOA73, which induced 73% larval mortality in *P. xylostella* after 120 h [65]. These findings may be linked to the presence of genes encoding Cry proteins in *P. popilliae* [36] and *Paenibacillus* sp. Kb-26 [66]. Additionally, genetic modification of *P. polymyxa* with the Cry1C gene has been shown to significantly increase its efficacy against lepidopteran pests [81]. Moreover, *Paenibacillus* is recognized for its dual function as an insecticidal agent and plant growth promoter [82]. However, specific evidence for *P. persimilis* remains limited, emphasizing the relevance of the present findings.

Molecular identification based on partial 16S rRNA gene sequences (~600 bp) allowed tentative assignment of the isolates to the families Bacillaceae, Planococcaceae, and Paenibacillaceae, and to the genera *Priestia*, *Lysinibacillus*, *Paenibacillus*, and *Peribacillus*. However, identity values less than 95% for several strains limit definitive taxonomic classification at the genus or species level [83,84]. Accordingly, conservative terminology has been used throughout the manuscript. Despite these limitations, the molecular phylogeny suggests that these isolates appear to be affiliated with bacterial groups previously reported as entomopathogenic species, highlighting the need for further taxonomic and functional characterization.

In all assays, the treated larvae exhibited consistent phenotypic changes, including lethargy, cessation of feeding, loss of turgor, progressive darkening, and death. These symptoms are consistent with bacterial infection processes previously described in lepidopterans exposed to entomopathogenic bacteria. Similar manifestations have been reported for various genera, including *Bacillus*, *Pseudomonas*, *Lysinibacillus*, *Serratia*, *Chromobacterium*, *Xenorhabdus*, and *Photorhabdus* [32,51,85,86,87,88], with members of the Bacillaceae family being the most extensively studied [30].

The relevance of these findings lies in the need to identify alternatives to the conventional use of *B. thuringiensis*, particularly in light of reports of resistance in some insect populations. Globally, several Gram-positive bacteria of the order Bacillales, including *Bacillus*, *Lysinibacillus*, and *Paenibacillus*, have demonstrated significant potential in biological pest control [27]. Moreover, the ability of many of these bacteria to form endospores represents a key advantage for persistence and viability under adverse environmental conditions [89].

Although the evaluated isolates induced significant larval mortality under controlled laboratory conditions, their applicability beyond controlled laboratory conditions remains uncertain. Additional studies are needed to assess their ecological performance, environmental stability, and effects on nontarget organisms under field conditions [76,90,91].

In this study, the evaluated native bacterial strains induced significant larval mortality under laboratory conditions, with concentration- and exposure-dependent mortality patterns. These findings provide preliminary evidence of entomopathogenic activity associated with bacterial exposure and suggest that some native isolates should be considered for future investigations.

However, the present results should be interpreted cautiously, as the efficacy of these isolates under field conditions remains to be confirmed. In addition, the mechanisms potentially associated with larval mortality were not investigated in this study, highlighting the need for further research to characterize the entomopathogenic activity of these isolates.

Overall, this study contributes to the understanding of native entomopathogenic bacteria associated with olive agroecosystems and provides baseline information for future investigations related to the management of *P. persimilis*.

## 4. Materials and Methods

### 4.1. Study Area and Sampling for the Detection of Entomopathogenic Bacteria

Soil samples were collected from olive plantations located in the district of La Yarada–Los Palos, province and department of Tacna, Peru, a representative arid agroecosystem dedicated to olive cultivation.

Sampling was conducted using a targeted design with spatial replication. Five independent sampling points were selected within the study area and considered independent experimental units. At each site, a composite soil sample of approximately 100 g was obtained from five subsamples collected at a depths of 10–20 cm, following a zigzag sampling pattern to capture spatial soil heterogeneity and ensure sample representativeness, in accordance with standardized environmental sampling guidelines [92].

The samples were placed in sterile polypropylene bags, properly labeled, and transported under refrigerated conditions (4 °C) to the Microbiology Laboratory of the Faculty of Sciences, Universidad Nacional Jorge Basadre Grohmann, where they were processed within 24 h after collection [93,94].

### 4.2. Isolation of Native Entomopathogenic Bacteria

For each soil sample, 10 g of soil was homogenized in 90 mL of sterile peptone water to obtain the initial inoculum suspension. Two independent serial dilution series were prepared up to 10^−5^. In the first dilution series, aliquots from the two final dilutions were surface-inoculated onto tryptic soy agar (TSA) and nutrient agar (NA) plates for the isolation of culturable heterotrophic bacteria.

In the second dilution series, the suspensions were subjected to heat treatment in a water bath at approximately 70–80 °C for 15 min to promote the selective isolation of endospore-forming bacteria. Subsequently, 100 μL aliquots were spread onto nutrient agar (NA) supplemented with fluconazole to inhibit fungal growth. All plates were incubated at 28–30 °C for 72 h [95,96].

### 4.3. Morphological and Microscopic Characterization of the Isolates

After incubation, individual bacterial colonies exhibiting distinct morphological characteristics were selected during the preliminary screening and purified by successive streaking on tryptic soy agar (TSA) plates to obtain axenic cultures. Macroscopic characterization of the purified isolates was performed based on colony morphology, including pigmentation, shape, texture, margin characteristics, elevation, and opacity. Subsequently, microscopic characterization was carried out by Gram staining.

Each purified isolate was subsequently inoculated into TSA slant vials, assigned a unique identification code, and stored at 5 °C for short-term preservation. Finally, all strains were cryopreserved in 30% glycerol solution and stored at −80 °C for long-term preservation [97].

### 4.4. Determination of Laboratory-Scale Entomopathogenic Activity of Native Bacteria

#### 4.4.1. Larval Collection

Larvae of *P. persimilis* used in the bioassays were collected at the second instar stage (L2) from olive plantations between 04:00 and 09:00 h to minimize thermal stress and preserve viability. Larvae were transported to the laboratory in transparent plastic containers covered with tulle fabric secured with an elastic band to allow ventilation and prevent escape. Precise larval instar determination was performed in the Entomology Laboratory, given the limited information available on *P. persimilis*. The larvae were subsequently transferred to the Microbiology Laboratory of the Faculty of Sciences, Universidad Nacional Jorge Basadre Grohmann, for use in bioassays [55,93].

#### 4.4.2. Inoculum Preparation

The isolated strains were reactivated in Luria–Bertani (LB) broth and incubated at 30 °C for 20 h. Subsequently, the cultures were streaked onto nutrient agar (NA) plates and incubated at 30 °C for an additional 20 h. Thereafter, three loopfuls of bacterial biomass were inoculated into nutrient broth and incubated at 30 °C for 24 h at 150 rpm to promote bacterial propagation and inoculum production.

Following incubation, the cultures were centrifuged at 8000 rpm for 15 min to remove the culture medium. The resulting pellets were washed three times with sterile phosphate-buffered saline (PBS; pH 7.0). Subsequently, the bacterial cells were resuspended in sterile distilled water, and the bacterial suspension used in the bioassays was adjusted by measuring optical density at 600 nm (OD600) to obtain an approximate concentration of 1 × 10^9^ CFU mL^−1^, following the methodology described by Eski et al. [98], with modifications.

#### 4.4.3. Pathogenicity Assay

For the pathogenicity bioassays, bacterial suspensions of the selected isolates were prepared. An initial exploratory screening assay was conducted using a concentration of 1 × 10^9^ CFU mL^−1^ to identify isolates exhibiting larvicidal activity. Subsequently, bioassays were performed using bacterial concentrations of 1 × 10^7^, 1 × 10^8^, and 1 × 10^9^ CFU mL^−1^, with three independent replicates per treatment. The bacterial suspensions were transferred into sterile 20 mL fine mist spray bottles for application for uniform application [99].

The diet consisted of fresh olive leaves collected from the field and superficially disinfected with 2% sodium hypochlorite for 2 min, followed by three rinses with sterile distilled water and drying with sterile absorbent paper [43]. Subsequently, the disinfected leaves were placed in suitable plastic containers following the methodology described by Eski et al. [98] with modifications.

Six previously disinfected olive leaves were placed in each container, and 2 mL of the bacterial suspension was uniformly sprayed onto both leaf surfaces [99,100]. The treated leaves were air-dried for approximately 1 h to promote adsorption of the bacterial inoculum onto the leaf surface. To maintain leaf moisture during the assay, a sterile moistened cotton plug was placed on the petiole of each leaf [58].

A commercial formulation of *B. thuringiensis* var. kurstaki (BIOSPORE 6.4% WP, wettable powder formulation, imported by FARMAGRO S.A., Lima, Peru) was used as the positive control, whereas the negative control consisted of olive leaves treated exclusively with sterile distilled water. Both controls were included in the preliminary and subsequent bioassays under the same experimental conditions.

Finally, ten second-instar (L2) larvae were transferred into each plastic container, which was covered with tulle fabric (15 × 17 cm) and secured with an elastic band to prevent larval escape while allowing ventilation, following the methodology described by Ninfa et al. [101]. For the preliminary screening assay, each isolate was evaluated using 10 second-instar (L2) larvae. Subsequently, the bioassays were conducted using three independent replicates per treatment (strain × concentration), with 10 larvae per replicate (*n* = 30 larvae per treatment combination). Larval mortality was recorded after 24, 48, 72, and 96 h of exposure.

### 4.5. Molecular Identification

Bacterial DNA extraction was performed exclusively from the isolates that exhibited the highest entomopathogenic activity. Prior to extraction, pure isolates were reactivated on LB agar and incubated at 35 °C for 24 h. Subsequently, genomic DNA was extracted from each isolate using the innuPREP Bacterial DNA Kit (Analytik Jena GmbH, Jena, Germany).

Amplification was performed by polymerase chain reaction (PCR) using the universal primers 27F (5′ AGA-GTTT-GATCMTGGCTCAG 3′) and 1492R (5′ TACGGYTACCTTGTTACGACTT 3′) [102,103,104]. PCRs were carried out in a final volume of 25 µL using Platinum Hot Start PCR Master Mix 2X (Invitrogen, Carlsbad, CA, USA), containing Platinum Taq DNA polymerase (Invitrogen, Carlsbad, CA, USA). The thermal cycling conditions included initial denaturation at 96 °C for 4 min, followed by 30 cycles of 94 °C for 30 s, 55 °C for 30 s, and 72 °C for 1 min, with a final extension of 10 min at 72 °C [104].

PCR products were subsequently quantified using a Qubit 4 fluorometer (Thermo Fisher, Waltham, MA, USA) and visualized by electrophoresis on 1% agarose gels. Amplicons showing visible bands were sent to Macrogen (Seoul, Republic of Korea) for sequencing.

### 4.6. Data Analysis

Larval mortality was recorded as the number of dead larvae relative to the total number of individuals exposed in each experimental replicate. Each treatment (strain × concentration) consisted of three independent replicates, with 10 larvae per replicate (*n* = 30 larvae per treatment combination), and mortality was evaluated after 24, 48, 72, and 96 h of exposure. The response variable consisted of proportions derived from binomial counts (dead/alive larvae); therefore, inferential analyses were based on models appropriate for binomial data.

The main inferential analysis was conducted using generalized linear models (GLMs) with a binomial distribution and logit link function, considering bacterial strain, inoculum concentration, and exposure time as explanatory variables. This approach allowed the simultaneous evaluation of multiple experimental factors affecting larval mortality, while accounting for the proportional and discrete nature of the response variable. Model selection was performed by comparing nested models with and without interaction terms using likelihood ratio tests. The final model was selected on the basis of statistical significance and biological interpretability.

Model fit was assessed by examining residual deviance and the dispersion parameter. Since no overdispersion was detected, the binomial model specification was retained. The GLM results are expressed as coefficients (β), standard errors (SEs), odds ratios (ORs), 95% confidence intervals (95% CIs), and significance values (*p* < 0.05).

Complementary exploratory comparisons among treatments were performed using nonparametric Kruskal–Wallis tests, followed by Dunn’s post hoc test with the Holm correction for multiple comparisons.

All the statistical analyses were performed in R (version 4.3.0).

## Figures and Tables

**Table 1:** Preliminary assays of the entomopathogenic activity of native bacterial isolates, expressed as percentage mortality at 96 h.

ID	Larvae			ID	Larvae		
	Alive	Dead	% Mortality		Alive	Dead	% Mortality
2M-2	8	2	20	4M-26	0	10	100
2M-4	2	8	80	5M-4	2	8	80
2M-5	4	6	60	5M-6	10	0	0
2M-9	10	0	0	5M-10	7	3	30
2M-16	8	2	20	5M-11	6	4	40
2M-23	8	2	20	5M-12	10	0	0
3M-5	5	5	50	5M-13	10	0	0
3M-19	4	6	60	5M-14	6	4	40
3M-22	1	9	90	5M-16	2	8	80
4M-1	0	10	100	5M-18	8	2	20
4M-5	3	7	70	5M-20	8	2	20
4M-12	8	2	20	5M-22	9	1	10
4M-13	9	1	10	6M-5	7	3	30
4M-14	4	6	60	6M-7	5	5	50
4M-21	8	2	20	6M-9	6	4	40
4M-23	8	2	20	6M-10	3	7	70
4M-24	6	4	40	6M-16	4	6	60
4M-25	8	2	20	6M-18	2	8	80
Positive control	0	10	100	Negative control	10	0	0

**Table 2:** Morphological characterization of native bacterial strains with entomopathogenic potential.

ID	Strain	Species	Microscopic Characteristics		Macroscopic Characteristics	
**3M-19**	Strain6	*Paenibacillus* sp. GCM	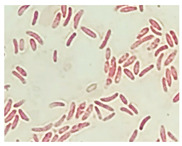	Rod-shaped, Gram-positive bacteria.	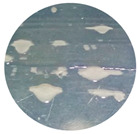	Texture: smooth Consistency: mucoid Appearance: shiny Elevation: convex Shape: fusiform Margin: entire Color: cream Transparency: opaque
**4M-14**	Strain7	*Lysinibacillus* sp. YLP	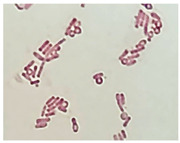	Palisade arrangement with large terminal spores; Gram-positive; cell size > 1 µm.	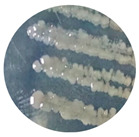	Texture: smooth Consistency: mucoid Appearance: shiny Elevation: flat Shape: circular Margin: entire Color: cream Transparency: opaque
**2M-5**	Strain5	*Priestia* sp. CCQ	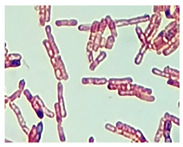	Gram-positive bacteria with endospores; cell size > 1 µm.	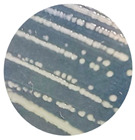	Texture: smooth Consistency: soft Appearance: dull Elevation: raised Shape: irregular Margin: undulate Color: cream Transparency: opaque
**4M-26**	Strain9	*Priestia* sp. JBG	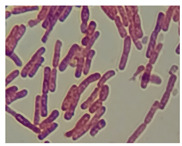	Diplobacilli with endospores; Gram-positive; cell size > 1 µm.	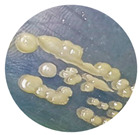	Texture: rough Consistency: soft Appearance: dull Elevation: crateriform Shape: circular Margin: entire Color: cream Transparency: opaque
**6M-18**	Strain4	*Peribacillus* sp. UNM	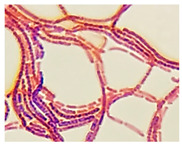	Long rod-shaped bacteria with endospores; Gram-positive; cell size > 1 µm.	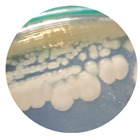	Texture: smooth Consistency: soft Appearance: dull Elevation: flat Shape: irregular Margin: undulate Color: cream Transparency: opaque
**4M-1**	Strain8	*Lysinibacillus* sp. ACM	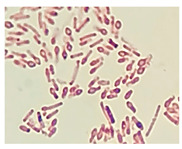	Palisade arrangement with large terminal endospores; Gram-positive; cell size > 1 µm.	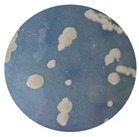	Texture: smooth Consistency: mucoid Appearance: shiny Elevation: raised Shape: irregular Margin: undulate Color: cream Transparency: opaque

**Table 3:** Molecular identification of native bacterial strains with entomopathogenic potential.

ID	Closest Match (NCBI)	Query Cover (%)	Identify (%)	Accession
2M-5	*Priestia* sp.	99%	83.69%	NR_164882.1
		99%	83.69%	NR115953.1
3M-19	*Paenibacillus* sp.	92%	92.73%	NR_113853.2
		92%	92.73%	NR_118012.1
		92%	92.57%	NR_024759.1
4M-1	*Lysinibacillus* sp.	68%	84.30%	NR_112569.1
		68%	84.30%	NR_112628.1
		68%	84.30%	NR_042072.1
4M-14	*Lysinibacillus* sp.	86%	87.38%	NR_112569.1
		86%	87.38%	NR_112628.1
		86%	87.38%	NR_042072.1
4M-26	*Priestia* sp.	96%	80.39%	NR_116873.1
		96%	80.39%	NR_117473.1
		96%	80.39%	NR_112636.1
6M-18	*Peribacillus* sp.	65%	90.1%	NR_117474.1
		65%	90.1%	NR_114919.1
		65%	90.1%	NR_112726.1

**Table 4:** Mean larval mortality (±standard error) of *P. persimilis* exposed to six native strains at different concentrations (10^7^, 10^8^ and 10^9^ UFC mL^−1^) evaluated at 24, 48, 72, and 96 h.

Strain	Concentration	Mean ± Standard Error			
		Mortality			
		24 h	48 h	72 h	96 h
*Lysinibacillus* sp. ACM Strain8	1 × 10^7^ CFU mL^−1^	3.33 ± 3.33	16.67 ± 6.67	66.67 ± 6.67	66.67 ± 6.67
	1 × 10^8^ CFU mL^−1^	26.67 ± 3.33	46.67 ± 6.67	73.33 ± 6.67	80 ± 5.77
	1 × 10^9^ CFU mL^−1^	23.33 ± 14.53	66.67 ± 13.33	86.67 ± 8.82	90 ± 10
*Peribacillus* sp. UNM Strain4	1 × 10^7^ CFU mL^−1^	60 ± 5.77	60 ± 5.77	76.67 ± 3.33	86.67 ± 3.33
	1 × 10^8^ CFU mL^−1^	60 ± 5.77	60 ± 5.77	76.67 ± 3.33	86.67 ± 3.33
	1 × 10^9^ CFU mL^−1^	66.67 ± 8.82	83.33 ± 8.82	86.67 ± 6.67	100 ± 0
*Priestia* sp. CCQ Strain5	1 × 10^7^ CFU mL^−1^	3.33 ± 3.33	10 ± 5.77	13.33 ± 6.67	20 ± 15.28
	1 × 10^8^ CFU mL^−1^	3.33 ± 3.33	10 ± 5.77	16.67 ± 8.82	30 ± 11.55
	1 × 10^9^ CFU mL^−1^	3.33 ± 3.33	10 ± 5.77	30 ± 5.77	40 ± 5.77
*Paenibacillus* sp. GCM Strain6	1 × 10^7^ CFU mL^−1^	30 ± 5.77	56.67 ± 3.33	70 ± 0	86.67 ± 3.33
	1 × 10^8^ CFU mL^−1^	36.67 ± 6.67	63.33 ± 8.82	80 ± 5.77	96.67 ± 3.33
	1 × 10^9^ CFU mL^−1^	60 ± 0	80 ± 5.77	93.33 ± 3.33	100 ± 0
*Lysinibacillus* sp. YLP Strain7	1 × 10^7^ CFU mL^−1^	50 ± 10	86.67 ± 3.33	90 ± 0	90 ± 0
	1 × 10^8^ CFU mL^−1^	56.67 ± 14.53	80 ± 10	86.67 ± 13.33	93.33 ± 6.67
	1 × 10^9^ CFU mL^−1^	53.33 ± 6.67	86.67 ± 3.33	96.67 ±3.33	96.67 ± 3.33
*Priestia* sp. JBG Strain9	1 × 10^7^ CFU mL^−1^	6.67 ± 6.67	56.67 ± 3.33	53.33 ± 3.33	60 ± 0
	1 × 10^8^ CFU mL^−1^	20 ± 11.55	66.67 ± 8.82	76.67 ± 3.33	86.67 ± 8.82
	1 × 10^9^ CFU mL^−1^	53.33 ± 17.64	93.33 ± 6.67	96.67 ± 3.33	96.67 ± 3.33

**Table 5:** Type II likelihood ratio tests were derived from the generalized linear model (GLM) with a binomial distribution and logit link function.

Factor	χ^2^ (LR)	df	*p*-Value
log10 (Concentration)	92.85	1	<0.001
Time	325.55	1	<0.001
Strain	527.53	5	<0.001
log10 (Concentration) × Time	3.85	1	0.049

**Table 6:** Binomial GLM with logit function applied to *P. persimilis* larval mortality as a function of concentration, time, and strain.

Term	β ± SE	OR (95% CI)	*p*-Value
Intercept	−3.73 ± 1.27	—	0.003
log10 (Concentration)	0.36 ± 0.16	1.44 (1.06–1.96)	0.021
Time	−0.004 ± 0.02	1.00 (0.96–1.04)	0.854
Strain5	−3.56 ± 0.22	0.03 (0.02–0.04)	<0.001
Strain6	−0.29 ± 0.19	0.74 (0.52–1.07)	0.114
Strain7	0.33 ± 0.20	1.39 (0.94–2.05)	0.096
Strain8	−1.24 ± 0.18	0.29 (0.20–0.41)	<0.001
Strain9	−0.71 ± 0.18	0.49 (0.34–0.71)	<0.001
log10 (Concentration) × Time	0.005 ± 0.003	1.01 (1.00–1.01)	0.051

**Table 7:** Bibliographic background on the entomopathogenic activity of *Priestia*, *Lysinibacillus*, *Paenibacillus*, *Bacillus*, and *Peribacillus* against *P. persimilis* and other lepidopteran species.

Country	Sample	Entomopathogenic Bacteria	Lepidopteran Species	Study Objective	Reference
Egypt	Soil	*Bacillus amyloliquefaciens*, *Pseudomonas putida*, and *Bacillus subtilis*	*Palpita unionalis*	74% larval mortality	[12]
Egypt	Soil	*B. thuringiensis*	*Spodoptera littoralis*	96.7% mortality within 3 days at 4 μg/g of crystal protein	[54]
Turkey	Dead insects	*Bacillus simplex*	*Galleria mellonella*	33% larval mortality every 24 h	[55]
Tunisia	Soil rhizospheric	*Paenibacillus polymyxa*	*P. unionalis*	Up to 30.6% mortality over 7 days	[56]
Tunisia	Gut	*Lysinibacillus* sp.	*Ephestia kuehniella*	56.7% mortality over 7 days	[57]
Egypt	Gut	*Priestia megaterium*	*Phyllocnistis citrella*	100% mortality within 6 days	[58]
Spain	Cotton leaves	*P. megaterium*	*S. littoralis*	LC50 of encapsulated Vip3Ag4 protein under UV exposure: 479.0 ng/cm^2^ after 7 days	[59]
Japan	Ant foregut	*Bacillus sphaericus* A3-2	*Spodoptera litura*	500 ng of sphaericolysin	[60]
Japan	Plants	–	*Palpita nigropunctalis*	Laboratory rearing on leaves	[61]
Argentina	Soil	*Bacillus toyonensis* biovar *thuringiensis*	*Cydia pomonella*	54.2 ± 17.7% mortality after 5 days with 5 μg/mL spore–crystal suspension	[62]
India	Lepidopteran larval cadavers	*Lysinibacillus fusiformis* FCC12	*Spodoptera* spp. and *Pectinophora gossypiella*	90% mortality	[63]
India	Gut	*Priestia flexa* and *Bacillus safensis*	*Biston suppressaria*	Pesticide resistance	[64]
Korea	Soil	*Paenibacillus elgii* HOA73	*Plutella xylostella*	73% larval mortality after 5 days of incubation	[65]
Sudan	Soil	*Paenibacillus* sp.	*H. armigera*	86.66% larval mortality after 15 days of incubation	[66]
Uruguay	INIA bacterial collection	*Lysinibacillus xylanilyticus*	*Argyrotaenia sphaleropa*	66% mortality on day 7 after exposure to 75 μL spore suspension	[67]
Brazil	Commercial product	*B. thuringiensis* subsp. *aizawai* GC-91	*S. eridania*	100% mortality at 1.25, 2.50, and 5 g/L after 7 days	[68]
Algeria	–	–	*Palpita vitrealis*	First record	[14]
United States	–	–	*P. persimilis*	Occurrence report	[15]
Uruguay	–	–	*P. persimilis*	First record	[6]
Peru	–	–	*P. persimilis*	Occurrence report	[52]
Several South American countries	–	–	*P. persimilis*	First record	[53]

## Data Availability

The data are available in this article.
